# Editorial: Decoding endurance performance: from theory to practice

**DOI:** 10.3389/fspor.2025.1643807

**Published:** 2025-06-25

**Authors:** Diego Jaén-Carrillo, Santiago Alejo Ruiz-Alias, Felipe García-Pinillos

**Affiliations:** ^1^ Department of Sport Science, University of Innsbruck, Innsbruck, Austria; ^2^ Department of Physical Education and Sport, University of Granada, Granada, Spain; ^3^ Sport and Health University Research Center (iMUDS), Parque Tecnologico de la Salud, Granada, Spain; ^4^ Department of Physical Education, Sport and Recreation, Universidad de La Frontera, Temuco, Chile

**Keywords:** testing, biomechanics, physiology, running, cycling, swimming

## Abstract

Endurance performance results from a complex interaction of physiological, biomechanical, psychological, and perceptual factors. Despite decades of research, transforming theoretical models into effective, individualized strategies for athletes remains a key challenge. This Research Topic, Decoding Endurance Performance: From Theory to Practice, brings together interdisciplinary studies that advance our understanding of how runners prepare, adapt, and perform in real-world conditions.

**Editorial on the Research Topic**
Decoding endurance performance: from theory to practice

The four original contributions included in this collection explore diverse yet complementary domains: biomechanical awareness, mental preparation, sex-specific physiological adaptations, and training dose quantification. Together, they shed light on practical approaches to monitoring and optimizing endurance performance across the athlete spectrum.

## Summary of contributions

### Self-reported vs. actual foot strike patterns in runners

Vincent et al. investigated the accuracy of self-reported foot strike patterns among 710 endurance runners using motion capture and treadmill analysis. They found that only 42.7% of runners correctly identified their strike pattern, with rearfoot strikers particularly inaccurate. Notably, runners who were unaware of their foot strike pattern had the highest prevalence of recent running-related injuries (RRIs). Footwear characteristics, such as heel-to-toe drop, were associated with both foot strike awareness and injury incidence. This study highlights the importance of objective gait assessments and targeted feedback for injury prevention and training adaptation strategies.

### Mindfulness for psychological readiness in elite runners

Kelemen et al. examined the effects of a six-week group-based mindfulness intervention (MSPE) in elite middle- and long-distance runners. Compared to a control group, athletes who completed the mindfulness program reported improved flow states, reduced cognitive and somatic anxiety, and enhanced emotional regulation. These findings suggest that mindfulness training can be a valuable, scalable tool for optimizing mental readiness in high-performance settings. The intervention's sport-specific tailoring further strengthens its practical application for endurance athletes preparing for competition.

### Durability predicts road cycling success

Barsumyan et al. investigated durability—defined as maintaining performance under prolonged fatigue—in 14 well-trained amateur cyclists during home-based testing. Participants performed 5- and 20-min time trials before and after a fatiguing protocol (∼1,000 kJ work at 70%–80% of maximal effort). Findings indicated that successful cyclists exhibited only a ∼6.5% power drop in the 20-min test under fatigue, compared to ∼12.5% in less successful peers. Heart rate responses did not differ, prompting a consideration of incorporating durability assessments in remote training and performance evaluation.

### Machine learning in endurance physiology

Boudry et al. offered a mini-review of machine learning (ML) methods in endurance and exercise physiology, aimed at bridging physiology and data science for practitioners. They outlined key ML techniques—supervised, unsupervised, semi-supervised, and reinforcement learning—and highlighted applications such as predicting VO₂max from non-invasive data, modeling heart rate variability, and identifying physiological response patterns. The review calls for addressing ML limitations (e.g., model interpretability, overfitting, data quality) and advocates for collaborative tool development to facilitate ML adoption in exercise science.

## Overarching themes & practical implications

• Bridging awareness and performance: from gait recognition to durability metrics and ML-driven insights, these studies highlight the importance of enhancing athlete and coach understanding through objective feedback and innovative methods.
• Ecologically valid approaches: both the amateur cycling protocol and mindfulness study showcase field- and remote friendly interventions, illustrating the feasibility of “lab-to-field” translation.
• Personalization and precision: with variability across foot strike awareness, psychological readiness, durability, and physiological traits, adopting personalized strategies is increasingly necessary. ML offers promise for crafting individualized profiling and adaptive training plans.
• Towards integrated monitoring frameworks: these papers represent foundational elements, that is, biomechanical, psychological, physiological, computational, for building integrated athlete-monitoring systems that support sustainable endurance performance.

## Conclusion

The four contributions presented in this Research Topic offer a comprehensive view of endurance performance, combining theoretical depth with practical, coach-friendly tools. They progress our understanding of how athletes perceive and sustain performance, cope mentally, and can be profiled and supported through emerging technologies.

We thank all authors and reviewers for their work. Continued efforts to blend objective feedback, mental training, durability metrics, and computational methods will be essential for advancing endurance sport science and its application.

